# The Addition of Marigold Extract to the Diet Improved the Performance of Laying Hens in the Late Laying Period by Increasing Their Antioxidant Capacity, Lipid Metabolism, and Microbial Composition

**DOI:** 10.3390/antiox14091126

**Published:** 2025-09-17

**Authors:** Qiyue Yang, Keying Zhang, Jianping Wang, Shiping Bai, Qiufeng Zeng, Huanwei Peng, Yadong Mu, Yue Xuan, Shanshan Li, Xuemei Ding

**Affiliations:** Key Laboratory of Animal Disease-Resistant Nutrition, Ministry of Education, Institute of Animal Nutrition, Sichuan Agricultural University, Chengdu 611130, China; yangqiyue@stu.sicau.edu.cn (Q.Y.); zkeying@sicau.edu.cn (K.Z.); wangjianping@sicau.edu.cn (J.W.); shipingbai@sicau.edu.cn (S.B.); zqf@sicau.edu.cn (Q.Z.); phw@sicau.edu.cn (H.P.); 72097@sicau.edu.cn (Y.M.); 71128@sicau.edu.cn (Y.X.); 71449@sicau.edu.cn (S.L.)

**Keywords:** laying hen, marigold extract, antioxidant, lipid metabolism, microbial composition

## Abstract

The decrease in the production performance of laying hens during the later laying stage can be attributed to multiple factors, chief among them being oxidative stress and disrupted lipid metabolism. Quercetagetin, the active component of marigold extract, is a flavonoid whose polyhydroxy structure has greater antioxidant capacity than other flavonoids. In this study, we determined whether adding marigold extract to the diet can improve the antioxidant capacity and lipid metabolism of late-laying hens to increase their performance. In total, 800 *Lohmann* laying hens (45 weeks old) were randomly assigned to five treatment groups, each consisting of eight replicates (20 hens per replicate). Throughout the experiment, which lasted 24 weeks, the hens were fed diets containing 0, 200, 400, 600, or 800 mg/kg marigold extract. The results of the study showed that the addition of marigold extract to the feed significantly increased the egg production rate and qualified egg rate and reduced the feed-to-egg ratio; it also reduced the levels of oxidized products in eggs, serum, and liver, and improved the antioxidant capacity of the organism. Mechanistic studies showed that marigold extract could activate the *Keap*1-*Nrf*2 signaling pathway and up-regulate the gene expression of *CAT*, *SOD*, *GPX*, and *Nrf*2. In addition, marigold extract increased HDL and VLDL content in the liver, decreased TC and LDL content, and alteration of the cecal microbial composition. In conclusion, marigold extract showed good application value and potential as a safe and effective antioxidant additive in the late laying stage of laying hens.

## 1. Introduction

During egg farming, a reduction in the productivity of laying hens in the late stage of laying [1] can lead to considerable economic losses. Multiple factors contribute to decreased performance in laying hens, including reduced antioxidant levels [2], accumulation of liver fat [3], gut microflora [4], and impairments in the functions of the digestive and reproductive systems with age [5,6].

With metabolism, reactive oxygen species (ROS) are constantly produced [7]. When endogenous antioxidants and free radicals in the organism, peroxides, fail to reach a state of equilibrium between them, oxidative stress occurs [8]. Oxidative stress harms the organism, causing multiple pathologies and impaired growth in animals [9], such as congestive heart failure [9], degradation of meat quality [10], and the production of toxic compounds in eggs [11]. In poultry, poor management conditions, low nutritional levels, and poor environments can lead to oxidative stress [12], whereas oxidation in the body can be controlled by regulating the diet. Antioxidant feed additives reduce heat-stress-induced oxidative damage in poultry [13].

The metabolic health and reproductive performance of poultry are strongly affected by excessive fat deposition [14], and when laying hens enter the late laying stage, they are susceptible to lipid metabolism disorders, leading to liver fat accumulation and reduced egg quality [15]. The liver is the primary metabolic center of the body and regulates both lipid and glucose homeostasis [16]. Total cholesterol (TC) is transported to the liver for lipid metabolism and uptake by hepatocytes. An imbalance in hepatic lipid metabolism increases TC synthesis, decreases lipid droplet catabolism, and impairs secretion of very-low-density lipoproteins (VLDL), which leads to pathologic aggregation of TC in hepatocytes [17], inducing diseases. Therefore, balanced lipid metabolism is crucial for maintaining organismal health.

A healthy gut microbiota is important for adapting to the living environment [18,19] of an animal. Late-laying hens have weak resistance to internal and external environmental influences, and their gut microbiota is susceptible. Excessive free radical production and reduced antioxidant capacity caused by oxidative stress decrease the diversity of the intestinal microbiota and alter its microbial composition [20,21]. The cecal microbiota is significantly associated with laying performance, which highlights the importance of microbial homeostasis for optimal productivity and reproductive health in hens [22,23].

Marigold extract is particularly rich in quercetagetin (QG), a distinctive polyhydroxylated flavonoid whose antioxidant potential has been proven to surpass that of other known flavonoids. These flavonoids have a wide range of physiological activities, including antioxidant, anti-inflammatory, and antimicrobial properties.

The phenolic components of flavonoids can inhibit the formation of adipose tissues [24,25,26]. Clinical evidence indicates that polyphenols, especially highly effective antioxidants like QG, can mitigate oxidative stress-mediated pathogenesis, thereby preventing or delaying the progression of chronic diseases [27]. Studies in mice have demonstrated that QG’s antioxidant capacity can enhance pancreatic islets’ function [28]. The addition of QG to the diet has been reported to promote the growth of broiler chickens, enhance the antioxidant capacity, and improve intestinal health [29]. Moreover, QG has been demonstrated to reduce the diarrhea rate of piglets and improve their growth performance and immune function [30].

Many studies have assessed other flavonoids, but few studies have determined the effect of marigold extract on laying hens in the later stages of laying. Therefore, in this study, we investigated whether marigold extract can increase the production performance in late-laying hens by enhancing the antioxidant capacity of the hens and regulating their lipid metabolism and microbial composition.

## 2. Materials and Methods

### 2.1. Birds and Experimental Design

The experimental protocol was approved by the Institutional Animal Care and Use Committee of Sichuan Agricultural University (Ethics Approval Code: SICAUAC202110-2; Chengdu, China). In total, 800 reared laying hens (315 days old) were selected. The hens were fed basal rations for two weeks, after which they were randomly divided into five treatment groups, with eight replicates for each treatment and 20 chickens for each replicate. During the experimental period, which lasted 24 weeks, each experimental group was supplemented with 0, 200, 400, 600, or 800 mg/kg marigold extract in their basal diet. The marigold extract contained quercetin as the primary active component (≥80% purity; Hebei Chenguang Biotechnology Group Co., Hebei, China). The basal rations consisted of corn-soybean meal, with nutrient levels adjusted to comply with both NRC (1994) [31] and NY/T 33-2004 specifications [32]. The formulation and nutritional composition of the basal diets are presented in Table 1. This experiment was performed at the test base of the Ya’an Animal Nutrition Research Institute of Sichuan Agricultural University. All treatments were randomly assigned and evenly spread in the coop, and all chickens were raised in cages (45.5 cm × 42 cm × 46 cm) with four chickens per cage, five adjacent cages are one repetition. Laying hens were fed mash feed throughout the experiment and were exposed to 16 h of light per day, with free access to food and water. The first meal was provided at 9:00 a.m., and after no abnormalities in the pens or chickens were observed, the second meal was provided at 3:00 p.m. Following daily observation of pen conditions and the health status of the birds, eggs were collected and weighed, and regular pen sanitation was maintained.

### 2.2. Laying Performance and Egg Quality

Daily records were maintained for egg production (total count) and egg mass (total weight) across all experimental periods. The average daily feed intake (ADFI) of hens was recorded based on the difference between the amount of feed added and the amount of residual feed added every week in replicates to calculate the feed–egg ratio, egg production rate, and average egg weight. Various parameters were calculated using the following formulae: egg production rate (%) = (number of eggs/number of hens) × 100; the qualified egg rate (%) = (number of qualified eggs/number of hens) × 100; the number of qualified eggs = the total number of eggs − the number of large eggs (>70 g), small (<50 g), broken, soft-shelled, sandy-shelled, dirty, and deformed eggs; average egg weight = total egg weight/number of eggs produced; feed-to-egg ratio (g/g) = total feed intake/total egg weight.

At the 12th and 24th weeks, five eggs per replicate were randomly selected for quality assessment, with 40 eggs per treatment group for analysis. The following egg quality traits were determined: eggshell strength, yolk color, Haugh unit, and albumen height. Eggshell strength was measured using an eggshell strength meter (model: EFR-01, ORKA, Venice, CA, USA). Yolk color, Haugh units, and albumen height were determined using an egg-quality automatic analyzer (model: ETP-01, ORKA, USA).

### 2.3. Serum Biochemical Parameters

At the 24th week, one hen per replicate (selected based on proximity to mean body weight) underwent jugular venipuncture. Blood samples were aliquoted into three collection tubes, allowed to clot, and centrifuged at 3000 r/min for 10 min. Serum samples were transferred to sterile EP tubes and stored at –20 °C until analysis. Total protein (TP), albumin [27], alanine aminotransferase (ALT), aspartate aminotransferase (AST), alkaline phosphatase (ALP), uric acid (UA), TC, triglyceride (TG), high-density lipoprotein (HDL), and low-density lipoprotein (LDL) levels were measured using a fully automated biochemical analyzer (HITACHI 7180, Tokyo, Japan), and VLDL contents were detected using an ELISA kit (Jiangsu Meimian Industrial Co., Ltd., Yancheng, China).

### 2.4. Liver Lipid Metabolism Parameters

At the 24th week, the blood-collected laying hens were slaughtered, with liver specimens immediately frozen at –80 °C. The TC, TG, HDL, LDL, and VLDL contents were measured using ELISA kits (Jiangsu Meimian Industrial Co., Ltd., Yancheng, China).

### 2.5. Antioxidants of the Body

At the 12th and 24th weeks, three eggs of normal morphology and size were collected from each replicate, and the egg whites and yolks were separated, mixed separately, and the samples were frozen at −20 °C for measuring antioxidant indicators in egg whites and yolks. The indices measured included 2,2-Diphenyl-1-picrylhydrazyl (DPPH) radical scavenging activity, restoring power, protein carbonyl content, and malondialdehyde (MDA) content.

At the 24th week, the laying hens from which blood was collected were slaughtered, and the liver samples were frozen at −80 °C for preservation. The total antioxidant capacity (T-AOC), superoxide dismutase (SOD) activity, and MDA content in the serum and liver were measured, along with the DPPH radical scavenging activities, reducing power, and protein carbonyl groups in the liver.

DPPH radical scavenging activity: the free radical scavenging power was determined following the method described by Zhang [33] with some modifications. A certain amount of sample was weighed, diluted nine times with anhydrous ethanol, prepared as a 10% homogenate, centrifuged at 3500 r/min for 10 min, and measured. The supernatant (100 µL) and DPPH (100 µL of 0.1 mmol/L DPPH prepared with 95% ethanol) (Sample A) were mixed thoroughly and left undisturbed for 30 min in the dark at room temperature. Absorbance readings were taken at 517 nm, and a self-controlled sample was prepared for each tube (the DPPH solution was replaced with 95% ethanol, a sample control). Additionally, a blank control was prepared for each batch of samples (the samples were replaced with 95% ethanol, a blank). DPPH radical scavenging activities (%) = (A blank − (A sample − A sample control)) × 100/A blank

Restoring power: this was performed following the methods described by Klompong [34], with some modifications. A total of 0.5 mL of 10% sample supernatant and 0.5 mL of 1% potassium ferricyanide solution were mixed thoroughly at 50 °C in a water bath for 20 min. Then, 0.5 mL of 10% trichloroacetic acid was added, and the mixture was vortexed and centrifuged at 6000 r/min for 6 min. Next, 0.5 mL of supernatant was collected, and 0.5 mL of double-distilled water and 0.1 mL of 0.1% ferric chloride were mixed well. Following mixing, samples were held at room temperature (25 °C) for 10 min, then absorbance was recorded at 700 nm.

Other antioxidant indices were measured following the protocols provided with the kits purchased: T-AOC, SOD, and MDA kits from Nanjing Jiancheng Bioengineering Institute (Nanjing, China). The protein carbonyl content was determined using a kit purchased from Beijing Suo Laibao Technology Co., Ltd. (Beijing, China).

### 2.6. RNA Isolation and Real-Time Quantitative PCR

The RNA extraction kit, mRNA reverse transcription kit, and real-time fluorescence quantification reagent required for the test were purchased from Takara (Dalian, China). All operations were performed strictly following the reference manual. The PCRs were performed using the CFX96 Real PCR System. A 384-well plate was used for the experiment, three parallel wells were used for each sample, β-actin was selected as the reference gene for normalization of the quantitative PCR data, and the design of the genes and primers is shown in Table 2.

### 2.7. Cecal Content

At the 24th week, the laying hens from which blood was collected were slaughtered, the cecum was removed, and the cecal chow was extruded, collected in EP tubes, and frozen at −80 °C for testing. Cecal microbial 16S rRNA analysis, including species relative abundance, microbial α diversity, and PCOA analysis, was performed by Rhonin Biosciences (Chengdu, China).

### 2.8. Statistical Analysis

Data analysis (SPSS 25.0) utilized one-way ANOVA and linear and quadratic regression analyses as well as Duncan’s multiple range test. Results are expressed as mean and SEM; *p* < 0.05 denotes significance.

Quadratic regression analysis was performed using SPSS 25.0 to determine the optimal supplemental level of marigold extract for each response variable. The model was fitted using the Polynomial Regression procedure by adding the squared term of the supplemental level (X^2^) as an independent variable. The regression model was formulated as Y = β_0_ + β_1_X + β_2_X^2^, where Y represents the response variable, X is the supplemental level of marigold extract (mg/kg), and β_0_, β_1_, β_2_ are the regression coefficients. The optimal dose was calculated as X_opt_ = −β_1_/(2β_2_) for models demonstrating a significant quadratic effect (*p* < 0.05 for the X^2^ term) and a concave downward relationship (indicated by β_2_ < 0). The goodness of fit for each model was assessed using the coefficient of determination (R^2^).

## 3. Results

### 3.1. Production Performance

Supplementing the rations with 400, 600, or 800 mg/kg marigold extract significantly improved the egg production rate and egg quality rate (*p* < 0.05) and significantly decreased the feed–egg ratio (*p* < 0.05) during the experimental period, and all showed significant linear and quadratic variations (*p* < 0.05) (Table 3). Dietary supplementation with marigold extract did not significantly affect the average feed intake or egg weight of laying hens (*p* > 0.05).

The quadratic regression model for the relevant data is as follows:

Egg production rate: Y = 86.90 + 0.0119X − 0.000014X^2^, R^2^ = 0.987, *p* < 0.001, optimal additive dose 425 mg/kg.

Qualified egg rate: Y = 85.91 + 0.0137X − 0.000016X^2^, R^2^ = 0.984, *p* < 0.001, optimal additive dose 428 mg/kg.

Feed to egg rate: Y = 2.043 − 0.0002X + 2.03 × 10^−7^X^2^, R^2^ = 0.981, *p* < 0.001, optimal additive dose 493 mg/kg.

### 3.2. Egg Quality

No significant differences in egg quality were observed with marigold extract supplementation at 12 or 24 weeks (*p* > 0.05; Table 4).

### 3.3. Serum Biochemistry

The results in Table 5 show that supplementation with marigold extract did not significantly alter serum biochemical parameters at week 24 (*p* > 0.05).

### 3.4. Antioxidants of Eggs

Addition of marigold extract to the diet linearly reduced triglyceride content in egg yolks at 12 and 24 weeks (*p* < 0.05). Dietary marigold extract tended to reduce MDA content in egg yolks at week 12 with a significant quadratic curve change (*p* = 0.022) and significantly decreased it in week 24 egg yolks, and varies linearly and quadratically (*p* < 0.05) (Table 6). It significantly increased the DPPH radical scavenging activities of week 12 and 24 egg yolks and egg whites (*p* < 0.05) and significantly reduced the carbonyl content of proteins in week 24 egg yolks (*p* < 0.05). The data showed no differences in reducing power (A_700nm_) of egg yolk and egg white, and MDA content in egg white between treatments at weeks 12 and 24 (*p* > 0.05). In addition, there was no significant difference in protein carbonyl content at week 12 (*p* > 0.05).

The quadratic regression model for the relevant data is as follows:

Week 12 yolks MDA: Y = 4.60 − 0.0037X + 3.87 × 10^−6^X^2^, R^2^ = 0.844, *p* < 0.05, optimal additive dose 478 mg/kg.

Week 12 yolks DPPH: Y = 42.31 + 0.026X − 0.000026X^2^, R^2^ = 0.939, *p* < 0.05, optimal additive dose 500 mg/kg.

Week 12 egg white DPPH: Y = 22.56 + 0.026X − 0.000023X^2^, R^2^ = 0.968, *p* < 0.001, optimal additive dose 565 mg/kg.

Week 24 yolks MDA: Y = 4.47 − 0.0045X + 5.26 × 10^−6^X^2^, R^2^ = 0.971, *p* < 0.001, optimal additive dose 428 mg/kg.

Week 24 yolks DPPH: Y = 36.27 + 0.032X − 0.000031X^2^, R^2^ = 0.901, *p* < 0.05, optimal additive dose 516 mg/kg.

Week 24 protein carbonyls: Y = 0.191 − 0.0003X + 3.33 × 10^−7^X^2^, R^2^ = 0.974, *p* < 0.001, optimal additive dose 450 mg/kg.

Week 24 egg white DPPH: Y = 24.17 + 0.019X − 0.000016X^2^, R^2^ = 0.921, *p* < 0.05, optimal additive dose 594 mg/kg.

### 3.5. Antioxidants in the Serum and Liver

The marigold extract added to the diets significantly decreased the serum MDA levels at 12 weeks with significant linear and quadratic changes (*p* < 0.05; Table 7). A significant linear increase in serum SOD (*p* = 0.036) and liver SOD (*p* = 0.024) at 24 weeks was observed with increasing amount of marigold extract. Marigold extract significantly increased liver DPPH radical scavenging activity (*p* < 0.05). Supplementation with 400 and 600 mg/kg marigold extracts significantly decreased the liver protein carbonyl content (*p* < 0.05). In addition, the addition of marigold extract had no significant effect on 12-week serum T-AOC, SOD, and 24-week serum T-AOC, MDA, as well as on liver T-AOC, MDA, and reducing power (*p* > 0.05).

The quadratic regression model for the relevant data is as follows:

12-week serum MDA: Y = 8.76 − 0.011X + 0.000011X^2^, R^2^ = 0.808, *p* < 0.05, optimal additive dose 500 mg/kg.

12-week serum SOD: Y = 76.34 + 0.049X − 0.000048X^2^, R^2^ = 0.850, *p* < 0.05, optimal additive dose 510 mg/kg.

24-week liver SOD: Y = 3.56 + 0.005X − 0.000005X^2^, R^2^ = 0.886, *p* < 0.05, optimal additive dose 500 mg/kg.

24-week liver DPPH: Y = 92.63 + 0.008X − 0.000007X^2^, R^2^ = 0.968, *p* < 0.001, optimal additive dose 571 mg/kg.

24-week liver protein carbonyls: Y = 0.263 − 0.0004X + 4.23 × 10^−7^X^2^, R^2^ = 0.844, *p* < 0.05, optimal additive dose 473 mg/kg.

### 3.6. Antioxidant Gene Expression

**Figure 1:**
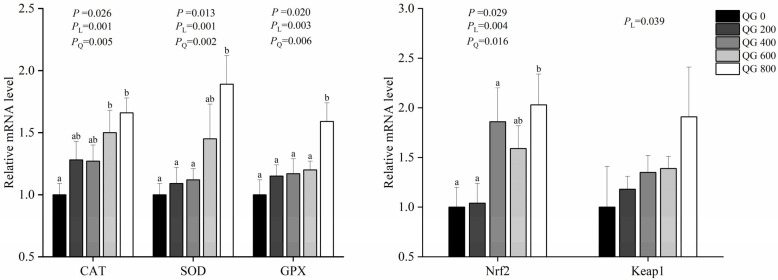
Effect of marigold extract on hepatic antioxidant activity in laying hens during the late laying period. Different lowercase letters indicate significant differences (*p* < 0.05), and no letters or identical letters indicate no significant difference (*p* > 0.05). The error bars represent the standard error of the mean (SEM).

The effect of marigold extract on the expression of antioxidant-related genes in the liver, and the results showed that 600 and 800 mg/kg of marigold extract significantly increased the mRNA expression of catalase (CAT) (*p* < 0.05; Figure 1). Additionally, 800 mg/kg marigold extract significantly increased the mRNA expression of SOD, glutathione peroxidase (GPX), and Nuclear Factor Erythroid 2-Related Factor 2 (Nrf2) (*p* < 0.05), and the mRNA expression of Kelch-like ECH-associated protein 1 (Keap1) also increased (*p* = 0.039).

### 3.7. Liver Lipid Metabolism

**Figure 2:**
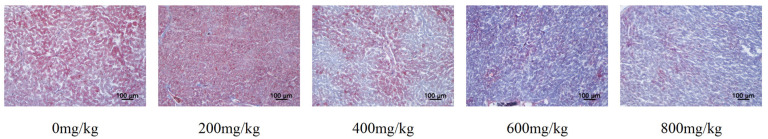
Effects of dietary marigold extract on the hepatic histomorphometry of H&E-stained sections from laying hens (100×).

The HE-stained liver sections exhibited extensive fat deposition in the control, 200 mg/kg, and 400 mg/kg groups, whereas treatment with 600 mg/kg and 800 mg/kg marigold extract considerably reduced hepatic fat accumulation (Figure 2). These findings indicate that optimal marigold extract supplementation alleviated liver steatosis in laying hens.

Dietary supplementation with 600 and 800 mg/kg marigold extract significantly elevated hepatic HDL levels (Table 8), as indicated by both significant linear and quadratic responses (*p* < 0.05). Adding 400, 600, and 800 mg/kg marigold extract significantly increased hepatic VLDL levels, with significant linear and quadratic changes (*p* < 0.05). The contents of TC (*p* = 0.042) and LDL (*p* = 0.024) in the liver decreased significantly and linearly (*p* < 0.05) as the marigold extract levels in the diet increased, but no significant effect was found on the TG content.

The quadratic regression model for the relevant data is as follows:

LDL: Y = 6.56 − 0.005X + 4.90 × 10^−6^X^2^, R^2^ = 0.766, *p* < 0.05, optimal additive dose 510 mg/kg.

HDL: Y = 18.97 + 0.016X − 0.000015X^2^, R^2^ = 0.945, *p* < 0.05, optimal additive dose 533 mg/kg.

VLDL: Y = 2.42 + 0.003X − 0.000003X^2^, R^2^ = 0.979, *p* < 0.001, optimal additive dose 500 mg/kg.

### 3.8. Microbial Composition and Function

**Figure 3:**
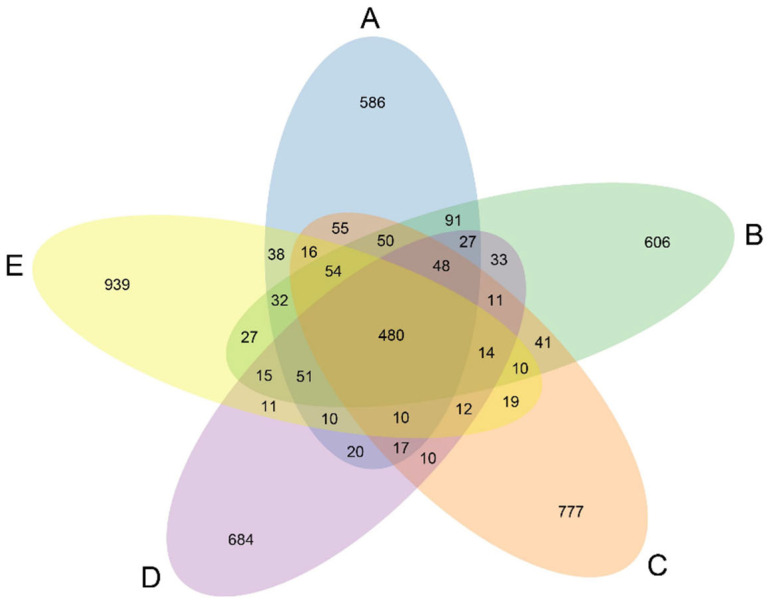
Venn diagram based on Operational Taxonomic Unit (OTU) classification. Each circle represents a group, and the number of circles and the overlapping part of the circle represent the number of. OTUs are common between the groups, and the number without overlap represents the number of OTUs unique to the group. (A) Control group, (B) 200 mg/kg marigold extract group, (C) 400 mg/kg marigold extract group, (D) 600 mg/kg marigold extract group, and (E) 800 mg/kg marigold extract group.

All groups shared 480 OTUs, but unique OTUs progressively increased across Groups A–E as the concentration of the marigold extract increased (Figure 3), indicating that the addition of marigold extract modulates the composition of the gut microbiota.

**Figure 4:**
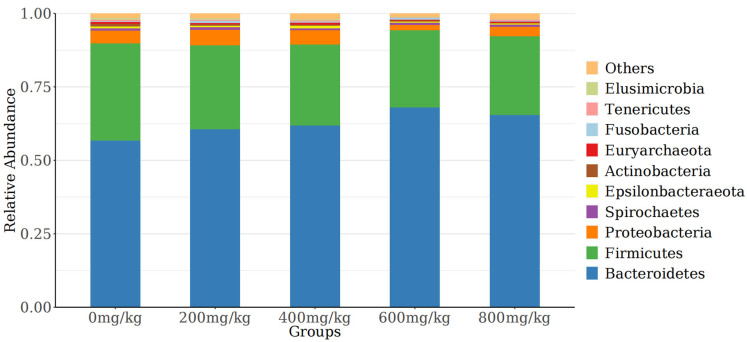
Histogram of the relative abundance of species at the phylum level.

**Figure 5:**
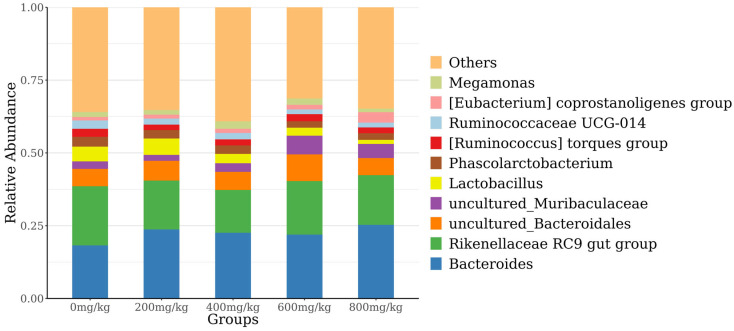
Histogram of the relative abundance of species at the genus level.

The 10 most abundant species at the phylum level were selected to generate a stacked histogram, while the other phyla were referred to as “others” (Figure 4). Among the phyla, *Bacteroidetes*, *Firmicutes*, and *Proteobacteria* had the highest relative abundances. The abundance of Bacteroidetes showed a quadratic curve with increasing marigold extract. Figure 5 displays the top 10 most abundant genera, with *Bacteroidales*, *Rikenellaceae_RC9_gut_group*, and *uncultured_Bacteroidales* being the predominant species.

**Figure 6:**
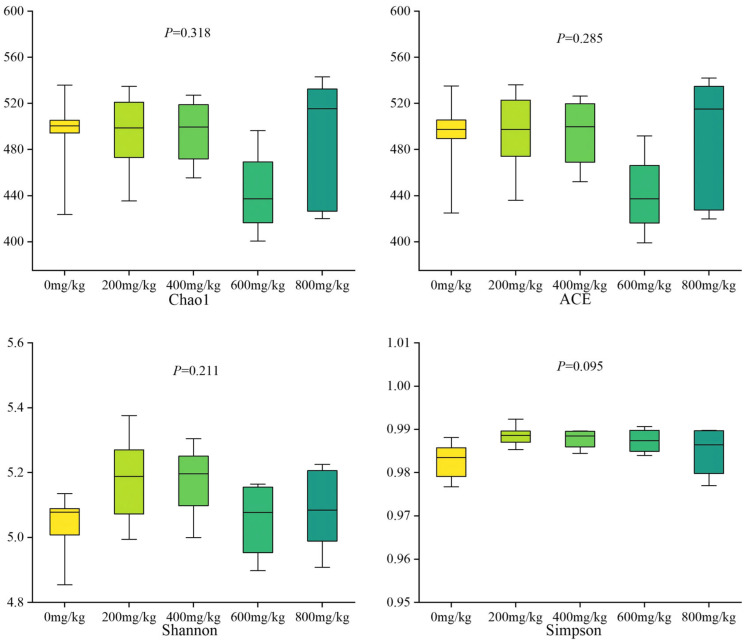
Effects of marigold extract on microbial alpha diversity in the cecum of laying hens. The error bars represent the standard error of the mean (SEM).

The results of the study on the effect of dietary addition of marigold extract on the α-diversity of cecum microorganisms in laying hens showed (Figure 6) that marigold extract had no significant effect on the diversity of cecum microbiota.

**Figure 7:**
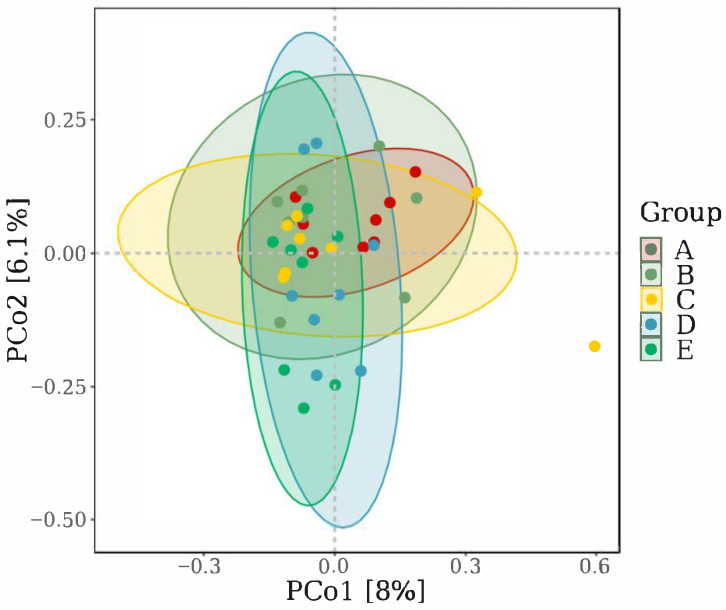
The beta diversity of the cecal microbiota was analyzed via PCoA. (A). Control group, (B) 200 mg/kg marigold extract group, (C) 400 mg/kg marigold extract group, (D) 600 mg/kg marigold extract group, and (E) 800 mg/kg marigold extract group.

As demonstrated by PCoA (Figure 7), there was no significant separation between the control and 200 mg/kg marigold extract groups (*p* > 0.05); 400 mg/kg or higher supplementation significantly altered the cecal microbiota composition in laying hens (*p* < 0.05).

## 4. Discussion

### 4.1. Dietary Supplementation with Marigold Extract Improves the Performance of Laying Hens in the Late-Laying Period

Laying hens, after experiencing intense metabolic changes during the peak laying period [3], enter the late laying period with severe metabolic disorders, including low body antioxidant levels [2], fatty liver [35], and impaired function of the reproductive system [36]. These factors collectively influence the production performance and egg quality of late-stage laying hens. Flavonoids, such as antioxidants, are important because of their physiological activities and ability to regulate lipid metabolism. Dietary supplementation with *Eucommia ulmoides* leaf extract enhances late-phase egg production performance in laying hens by inducing changes in blood metabolism and gut microbial populations [37]. Adding 400 mg/kg quercetin to the diet increased the laying rate and reduced the feed-to-egg ratio of laying hens [38]. Treatment with ipriflavone improved egg production in laying hens and ducks [39,40]. In this study, adding marigold extract to rations significantly increased the egg production rate and egg quality rate of laying hens in the late laying stage and reduced the feed–egg ratio. This indicated that marigold extract is beneficial to the performance of laying hens.

### 4.2. Addition of Marigold Extract to Feeding Rations Increases Antioxidant Levels in Eggs

Eggs are an inexpensive, nutritionally balanced food from animal sources. Through genetic regulation, laying hens prioritize the allocation of most of the energy and nutrients they consume from their diet to eggs, followed by their body [41,42]. Dietary antioxidant supplementation during the late laying period exhibits comparable effects [42]. Eggs contain abundant lipids that are prone to peroxidation, which decreases their nutritional value. As a terminal product of lipid peroxidation, MDA levels directly indicate the degree of oxidative damage in eggs. We found that the MDA content of egg yolk decreased significantly at 24 weeks (*p* < 0.05), which indicated that adding marigold extract to the diet of laying hens in the late laying stage can inhibit the lipid oxidation of eggs, which is consistent with the results reported by Simitzis et al. [43]. Some studies have reported that antioxidants are deposited. In eggs, improved antioxidant levels in the serum can also reduce lipid peroxidation [44]. Supplementing inulin containing antioxidant components in the diet can increase the activity of antioxidant enzymes and T-AOC in the serum of laying hens and decrease the content of MDA, thus improving the antioxidant status of eggs [45,46]. Marigold extract significantly reduced the serum MDA concentration at 12 weeks (*p* < 0.05) (Table 7), with a decrease in the overall MDA levels at 24 weeks. Although T-AOC and SOD in the serum did not differ significantly, they exhibited an increasing trend compared to the control group. These results suggest that after marigold extract was metabolized in laying hens, its antioxidant components probably decreased lipid peroxidation in eggs. Although marigold extract could not be directly deposited in egg yolk, elevated serum T-AOC and SOD levels, significantly lower MDA (*p* < 0.05, Table 7), enhanced DPPH radical scavenging activity in egg yolk and egg white, and lower yolk MDA and protein carbonyls were observed at 12 weeks (*p* < 0.05, Table 6). The underlying mechanism may be related to phenols with antioxidant properties in flavonoids.

### 4.3. Dietary Supplementation with Marigold Extract Improves Antioxidant Capacity in Late-Laying Hens

ROS produced by oxidative stress can disrupt redox balance, and aged laying hens are more susceptible to ROS and free radicals due to an imbalance in the redox system, which negatively affects their health [47,48]. As laying hens get older, the activities of several antioxidant enzymes in serum and liver tissue decrease [3], and antioxidant enzymes constitute the first line of defense against oxidative damage in the body. SOD catalyzes ROS conversion to hydrogen peroxide, then CAT converts it to water [49]. In this study (Table 7), dietary marigold extract supplementation increased SOD activity in the serum and liver of late-phase laying hens compared to the controls, suggesting that the former can mitigate oxidative stress. DPPH is a stable free radical, and its scavenging activity corresponds to the antioxidant capacity of the sample. Protein carbonyls are the hallmark products of protein oxidation. The significant increase in DPPH free radical scavenging activity and the significant decrease in protein carbonyl content in the liver in this study indicated that the antioxidant components in the marigold extract could effectively neutralize the excess free radicals produced in the body and mitigate oxidative damage.

*Keap*1-*Nrf*2 is the most important endogenous antioxidant signaling pathway [50]. The oxidative stress-sensing transcription factor Nrf2 is under the suppressive regulation of Keap1. When the body is in a normal physiological state, Nrf2 is tightly bound to Keap1. Under oxidative stress, Keap1 dissociates from Nrf2, which enters the nucleus and binds to antioxidant components, activating Nrf2 and initiating the gene expression of antioxidant enzymes such as *CAT*, *SOD*, and *GPX* [51]. Figure 1 results indicate that, compared to the control, the 600 and 800 mg/kg marigold extracts significantly increased the expression of the *CAT* and *SOD* genes (*p* < 0.05), and the 800 mg/kg marigold extract significantly increased the expression of the *GPX* and *Nrf2* genes (*p* < 0.05). These findings indicated that the inhibitory effect of Keap1 on Nrf2 was relieved and that the activity of Nrf2 increased. Studies investigating flavonoids in animal applications have shown the same results [52,53,54,55], indicating that supplementing marigold extract in the diet helps activate the antioxidant defense mechanism in laying hens in the late laying stage and increases the antioxidant capacity of the liver.

### 4.4. Dietary Supplementation with Marigold Extract Improves Lipid Metabolism in Late-Laying Hens

Caged hens in the late laying stages usually suffer from a high-energy diet and limited movement, leading to fatty liver [15,56], a common metabolic disease in older hens that leads to low hatchability and low egg production rates [57,58]. When lipid metabolism is disrupted, excess free fatty acids in adipose tissue are taken up by hepatocytes, converted into TG, and stored in the liver [59]. An increase in TG content is characteristic of fatty liver disease. This experiment demonstrated that dietary supplementation with 600 or 800 mg/kg marigold extract alleviated hepatic fat accumulation in laying hens (Figure 2). Quercetin, which is also a flavonoid compound, inhibits the apoptosis of adipocytes and adipogenesis and has antiobesity effects [60]. The application of quercetin in poultry production can decrease the fatty acid concentration of broiler chickens, improve meat quality [61], and reduce the TC and TG levels in egg yolk. Serum indicators often reflect the health state of the body; among these indicators, the levels of TC, TG, HDL, LDL, and VLDL reflect the lipid metabolism of animals. HDL transports excess TC from peripheral tissues back to the liver for metabolic elimination. The main function of LDL is to transport TC from the liver to peripheral tissues. We found no significant alterations in the serum lipid metabolism parameters of late-stage laying hens (Table 5). Further measurement of lipid metabolism-related indices in the liver of laying hens (Table 8) revealed that adding marigold extract to the diet significantly decreased TC (*p* = 0.042) and LDL (*p* = 0.024) in the liver linearly and significantly increased the level of HDL (*p* < 0.05). The significant increase in VLDL (*p* < 0.05) occurred probably due to the large amount of synthetic very-low-density lipoprotein yolk precursor (VLDLy) required by the liver during laying, which was then transported to the ovary as the main lipid and protein source of the yolk; this finding is consistent with the results reported by Guo et al. [62]. Studies have shown that marigold extract can inhibit the production of fat in the liver while promoting the synthesis of yolk precursors to improve the production performance of laying hens in the late laying stage.

### 4.5. Addition of Marigold Extract to the Ration Modulates the Intestinal Flora Structure of Laying Hens in the Late-Laying Period

The gut microbiota is crucial for maintaining organismal health [63,64], and cecal microorganisms are directly associated with the performance of laying hens [22]. The cecal microbial composition in this study, characterized by predominant Firmicutes, Bacteroidetes, and Proteobacteria, matches that of healthy laying hens reported in other studies [65,66]. Some probiotics involved in protein and polysaccharide degradation in Bacteroidetes can increase nutrient metabolism and egg production [67]. There are many pathogens in Proteobacteria, such as *Escherichia coli* and *Salmonella*. This study showed that marigold extract enriched *Bacteroidetes* but decreased *Firmicutes* and *Proteobacteria* in the cecal microbiome of laying hens. It should be noted that this study primarily revealed the changes in gut microbiota induced by marigold extract, while the underlying metabolic mechanisms remain to be elucidated. Studies have demonstrated that flavonoids undergo bidirectional interactions with the gut microbiota, where intestinal microorganisms actively participate in flavonoid metabolism and transformation [68]. In the intestinal microecosystem, flavonoids restrain harmful bacteria while promoting ecological balance. For instance, *Cyclocarya paliurus* flavonoids can restore dysregulated gut microbiota composition and functionality, consequently enhancing intestinal microecological homeostasis in mice [69]. This study indicates that dietary marigold extract supplementation during late lay may modulate gut microbiota composition in hens, enhancing health status and consequently improving production performance.

### 4.6. Determination of Optimal Dose by Quadratic Regression

Quadratic regression analysis was employed to quantitatively determine the optimal supplementation level. The results demonstrated highly significant (*p* < 0.05) quadratic relationships between marigold extract dose and all key indicators, including laying performance, antioxidant capacity, and lipid metabolism.

For egg production rate and qualified egg rate, the model calculated optimal dosages of 425 and 428 mg/kg, respectively. The optimal dosage for feed-to-egg ratio was 493 mg/kg. This indicates that from a production efficiency perspective, supplementation levels between 400 and 500 mg/kg already yield excellent results.

For indicators related to antioxidant activity and liver health, the optimal dosage of marigold extract is slightly higher, with many falling between 500 and 570 mg/kg. This suggests that achieving optimal bodily health may require a slightly higher supplemental dose.

Considering the dual objectives of enhancing production performance and improving animal health, and to avoid the cost waste of excessive supplementation, 400–600 mg/kg represents a safe, effective, and economical optimal dosage range.

## 5. Conclusions

In summary, adding marigold extract to the diet reduced the MDA and protein carbonyl contents in egg yolks and increased the DPPH radical scavenging activity in egg yolks and whites. The antioxidant capacity of marigold extract was reflected by the fact that adding marigold extract decreased the serum MDA content, increased serum and liver SOD activity, improved liver DPPH radical scavenging activities, decreased protein carbonyls in the liver, and regulated the expression of liver antioxidant genes. Second, marigold extract supplementation increased hepatic HDL and VLDL levels while reducing TC and LDL contents. Moreover, marigold extract modified gut microbiota composition and enhanced microbial diversity in the intestinal community. The results showed that marigold extract is an effective feed additive for laying hens in the later stages of life with a recommended dosage of 400–600 mg/kg.

## Figures and Tables

**Table 1:** Ingredient composition and nutrient levels of the basal diets (as-fed basis).

Ingredients	(%)
Corn	57.82
Soybean meal (CP, 43%)	23.80
Wheat bran	5.00
Soybean oil	1.75
Grainy limestone	6.90
Powdery limestone	2.30
CaHPO_4_	1.40
DL-methionine	0.10
NaCl	0.30
Choline chloride	0.10
Vitamin premix ^1^	0.03
Mineral premix ^1^	0.50
Total	100.00
Nutrient levels	
Metabolizable energy (Kcal/Kg)	2639.25
Crude protein	15.48
Calcium	3.95
Total phosphorus	0.60
Available phosphorus	0.34
Digestible lysine	0.80
Digestible methionine	0.34
Digestible threonine	0.59
Digestible tryptophan	0.17
Digestible methionine + cystine	0.62

^1^ Vitamin and mineral premixes are provided per kg of diet: VA 8000 IU, VD_3_ 1600 IU, VE 5 IU, VK_3_ 0.5 mg, VB_1_ 0.8 mg, VB_2_ 2.5 mg, VB_6_ 1.5 mg, VB_12_ 0.004 mg, calcium pantothenic 2.2 mg, folic acid 0.25 mg, niacinamide 20 mg, D-biotin 0.1 mg. Cu (CuSO_4_) 10 mg, Fe (FeSO_4_) 60 mg, Mn (MnSO_4_) 100 mg, Zn (ZnSO_4_) 60 mg, I (KI) 0.36 mg, Se (Na_2_SeO_3_) 0.3 mg.

**Table 2:** Design sequence of target genes and reference primers.

Gene Name	Primer Sequence (5′-3′)	Gene Bank ID
*β-Actin*	Forward: CCA GCC ATG TAT GTA GCC ATC CAG Reverse: ACG GCC AGC CAG ATC CAG AC	NM_205518
*CAT*	Forward: TAC TGC AAG GCG AAA GTG TT Reverse: GGA AAC AAC ATT GCA TCC CG	NM_001031215
*SOD*	Forward: GCA GGT GCT CAC TTT AAT CCT Reverse: CCA CAA GCT AAA CGA GGT CC	GGU28407
*GPX*	Forward: AAC GGC TTC AAA CCC AAC TT Reverse: GAC CAG ATG ATG TAC TGC GG	HM590226
*KEAP1*	Forward: CAT CGA CTG TTA CAA CCC CAT Reverse: CGG CGT ACA GCA GTA TGT T	KU321503.1
*NRF2*	Forward: CCC AAA ACT GCC GTA AGA GA Reverse: TGC CAT CTC TAG TTT GCT GC	NM_205117.1

**Table 3:** Effect of different levels of marigold extract on production performance (1–24 weeks).

Items	Marigold Extract Supplemental Levels (mg/kg)					SEM	*p*-Value		
	0	200	400	600	800		ANOVA	Linear	Quadratic
ADFI (g/day)	108.70	109.12	109.51	108.27	109.19	0.15	0.076	0.907	0.882
Egg production rate (%)	87.09 ^a^	88.52 ^ab^	89.87 ^bc^	89.77 ^bc^	90.39 ^c^	0.31	0.002	<0.001	<0.001
Qualified egg rate (%)	86.17 ^a^	87.54 ^ab^	88.71 ^b^	88.62 ^b^	89.45 ^b^	0.33	0.009	<0.001	<0.001
Average egg weight (g)	61.18	61.52	61.44	61.28	61.06	0.11	0.732	0.557	0.397
Feed to egg rate (g/g)	2.04 ^a^	2.01 ^ab^	1.98 ^b^	1.97 ^b^	1.98 ^b^	0.01	0.012	<0.001	<0.001

Different lowercase letters of peer shoulder notes indicate significant differences (*p* < 0.05), and no letters or identical letters indicate no significant difference (*p* > 0.05).

**Table 4:** Effect of different levels of marigold extract on egg quality.

Items	Marigold Extract Supplemental Levels (mg/kg)					SEM	*p*-Value		
	0	200	400	600	800		ANOVA	Linear	Quadratic
Week 12									
Albumen height (mm)	7.15	6.97	6.64	6.44	6.85	0.09	0.086	0.072	0.038
Haugh unit	83.04	82.34	79.55	78.72	81.32	0.59	0.098	0.092	0.050
Egg yolk color	5.38	5.80	5.31	5.58	5.90	0.08	0.109	0.174	0.291
Eggshell strength (kg/cm^2^)	4.58	4.67	4.88	4.62	4.65	0.06	0.575	0.838	0.497
Week 24									
Albumen height (mm)	6.90	7.07	7.06	6.94	7.11	0.08	0.927	0.621	0.875
Haugh unit	81.33	82.73	82.74	81.68	82.72	0.60	0.912	0.685	0.866
Egg yolk color	5.44	5.21	4.97	5.06	4.88	0.08	0.241	0.031	0.082
Eggshell strength (kg/cm^2^)	4.13	4.37	4.40	4.30	4.06	0.07	0.495	0.688	0.178

**Table 5:** Effect of different levels of marigold extract on serum biochemical of laying hens (week 24).

Items	Marigold Extract Supplemental Levels (mg/kg)					SEM	*p*-Value		
	0	200	400	600	800		ANOVA	Linear	Quadratic
ALT (U/L)	5.68	6.52	6.05	5.11	5.60	0.31	0.763	0.575	0.760
AST (U/L)	163.99	157.74	163.54	161.53	156.79	1.63	0.603	0.469	0.722
TP (g/L)	52.08	54.39	55.63	52.64	53.60	1.07	0.864	0.872	0.735
ALB (g/L)	17.95	19.22	17.56	17.82	17.53	0.26	0.346	0.302	0.555
GLB (g/L)	32.14	35.86	36.67	36.34	33.71	1.10	0.644	0.619	0.278
AKP U/L)	802.71	783.00	754.33	730.50	689.67	70.56	0.989	0.571	0.853
UA (μmol/L)	145.06	149.88	143.31	142.39	145.23	5.13	0.996	0.957	0.983
TC (mmol/L)	3.03	3.52	3.22	2.72	2.98	0.12	0.335	0.379	0.503
TG (mmol/L)	17.27	15.70	15.89	15.02	16.67	0.63	0.833	0.567	0.551
LDL (mmol/L)	1.49	1.64	1.42	1.38	1.50	0.05	0.526	0.513	0.774
HDL (mmol/L)	1.02	1.04	0.94	0.92	0.84	0.04	0.447	0.064	0.167
VLDL (mmol/L)	7.12	6.58	7.70	6.98	7.21	0.16	0.258	0.600	0.845

**Table 6:** Effect of different levels of marigold extract on the antioxidants of eggs.

Items	Marigold Extract Supplemental Levels (mg/kg)					SEM	*p*-Value		
	0	200	400	600	800		ANOVA	Linear	Quadratic
Week 12									
Triglyceride (mmol/g)	0.02	0.02	0.02	0.02	0.02	0.00	0.189	0.017	0.053
Yolks MDA (nmol/mg)	4.71	3.75	3.71	3.82	3.98	0.12	0.067	0.181	0.022
Yolks DPPH (%)	42.40 ^a^	42.34 ^a^	45.07 ^ab^	47.09 ^b^	44.26 ^ab^	0.60	0.050	0.051	0.059
Yolks restoring power (A_700nm_)	0.35	0.37	0.36	0.37	0.38	0.01	0.695	0.293	0.576
Protein carbonyls	0.18	0.16	0.16	0.18	0.14	0.01	0.447	0.273	0.518
Egg white MDA (nmol/mg)	1.50	1.53	1.55	1.66	1.76	0.06	0.641	0.123	0.280
Egg white DPPH (%)	22.77 ^a^	25.49 ^a^	28.77 ^b^	28.43 ^b^	28.3 ^b^	0.63	0.004	<0.001	<0.001
Egg white restoring power (A_700nm_)	0.14	0.14	0.14	0.14	0.15	0.00	0.924	0.928	0.776
Week 24									
Triglyceride (mmol/g)	0.02	0.02	0.02	0.02	0.02	0.00	0.391	0.045	0.138
Yolks MDA (nmol/mg)	4.53 ^a^	3.64 ^b^	3.75 ^b^	3.59 ^b^	3.42 ^b^	0.12	0.032	0.008	0.014
Yolks DPPH (%)	37.39 ^a^	42.49 ^b^	42.45 ^b^	44.05 ^b^	42.40 ^b^	0.75	0.043	0.025	0.010
Yolks restoring power (A_700nm_)	0.32	0.34	0.36	0.35	0.34	0.01	0.519	0.293	0.191
Protein carbonyls	0.17 ^a^	0.13 ^b^	0.13 ^b^	0.13 ^b^	0.12 ^b^	0.01	0.041	0.009	0.016
Egg white MDA (nmol/mg)	1.58	1.67	1.71	1.76	1.78	0.05	0.758	0.174	0.384
Egg white DPPH (%)	24.13 ^a^	27.26 ^b^	27.65 ^b^	27.71 ^b^	28.86 ^b^	0.51	0.036	0.005	0.010
Egg white restoring power (A_700nm_)	0.14	0.14	0.14	0.14	0.14	0.00	0.971	0.664	0.911

Different lowercase letters of peer shoulder notes indicate significant differences (*p* < 0.05), and no letters or identical letters indicate no significant difference (*p* > 0.05).

**Table 7:** Effect of different levels of marigold extract on the antioxidants of the body.

Items	Marigold Extract Supplemental Levels (mg/kg)					SEM	*p*-Value		
	0	200	400	600	800		ANOVA	Linear	Quadratic
12-week serum									
T-AOC (UmL)	0.66	0.72	0.70	0.79	0.66	0.02	0.235	0.594	0.299
MDA (nmol/mL)	8.54 ^a^	7.62 ^ab^	7.26 ^ab^	6.87 ^ab^	5.94 ^b^	0.28	0.047	0.002	0.008
SOD (U/mL)	84.69	86.76	86.48	92.16	87.19	1.14	0.312	0.201	0.283
24-week serum									
T-AOC (U/mL)	0.79	0.87	0.89	0.82	0.86	0.02	0.561	0.502	0.468
MDA (nmol/mL)	8.12	8.11	7.98	8.00	7.41	0.17	0.707	0.215	0.373
SOD (U/mL)	77.76	81.58	86.74	83.11	89.05	1.65	0.219	0.036	0.109
24-week liver									
T-AOC (U/mgprot)	0.10	0.11	0.11	0.11	0.11	0.00	0.993	0.887	0.949
MDA (nmol/mgprot)	0.49	0.44	0.54	0.52	0.52	0.02	0.661	0.378	0.664
SOD (U/mgprot)	3.61	3.85	4.53	4.82	4.83	0.21	0.242	0.024	0.069
DPPH (%)	93.32 ^a^	94.98 ^b^	94.79 ^b^	94.66 ^b^	95.04 ^b^	0.15	<0.001	0.003	<0.001
Restoring power (A_700nm_)	0.40	0.42	0.43	0.45	0.43	0.01	0.401	0.110	0.199
Protein carbonyls	0.26 ^a^	0.22 ^ab^	0.20 ^b^	0.18 ^b^	0.25 ^a^	0.01	0.005	0.341	<0.001

Different lowercase letters of peer shoulder notes indicate significant differences (*p* < 0.05), and no letters or identical letters indicate no significant difference (*p* > 0.05).

**Table 8:** Effect of different levels of marigold extract on the liver lipid metabolism (week 24).

Items	Marigold Extract Supplemental Levels (mg/kg)					SEM	*p*-Value		
	0	200	400	600	800		ANOVA	Linear	Quadratic
TG (mmol/L)	7.25	6.94	6.97	6.61	6.55	0.19	0.797	0.206	0.455
TC (mmol/L)	7.27	7.79	7.40	6.78	6.44	0.18	0.195	0.042	0.052
LDL (mmol/L)	6.52	5.92	6.16	5.84	5.15	0.18	0.175	0.024	0.071
HDL (mmol/L)	19.20 ^ab^	19.70 ^abc^	18.52 ^a^	22.72 ^c^	22.15 ^bc^	0.54	0.036	0.018	0.044
VLDL (mmol/L)	2.38 ^a^	2.74 ^ab^	3.21 ^b^	3.02 ^b^	3.10 ^b^	0.09	0.037	0.011	0.010

Different lowercase letters of peer shoulder notes indicate significant differences (*p* < 0.05), and no letters or identical letters indicate no significant difference (*p* > 0.05).

## Data Availability

The data presented in this study are available on request from the corresponding author. The data are not publicly available due to the containing information that could compromise the privacy of research participants.

## Abbreviations

TC	Total cholesterol
VLDL	Very-low-density lipoproteins
ADFI	Average daily feed intake
TP	Total protein
ALB	Albumin
ALT	Alanine aminotransferase
AST	Aspartate aminotransferase
ALP	Alkaline phosphatase
UA	Uric acid
TG	Triglyceride
HDL	High-density lipoprotein
LDL	Low-density lipoprotein
DPPH	2,2-Diphenyl-1-picrylhydrazyl
QG	Quercetagetin
OUT	Operational Taxonomic Unit
MDA	Malondialdehyde
T-AOC	Total antioxidant capacity
SOD	Superoxide dismutase
CAT	Catalase
GPX	Glutathione peroxidase
Keap1	Kelch-like ECH-associated protein 1
Nrf2	Nuclear Factor Erythroid 2-Related Factor 2
ROS	Reactive oxygen species
VLDLy	Very-low-density lipoprotein yolk

## References

[1] Tůmová E., Uhlířová L., Tůma R., Chodová D., Máchal L. (2017). Age related changes in laying pattern and egg weight of different laying hen genotypes. Anim. Reprod. Sci..

[2] Lin H., De Vos D., Decuypere E., Buyse J. (2008). Dynamic changes in parameters of redox balance after mild heat stress in aged laying hens (*Gallus gallus domesticus*). Comp. Biochem. Physiol. Part C Toxicol. Pharmacol..

[3] Gu Y.F., Chen Y.P., Jin R., Wang C., Wen C., Zhou Y.M. (2021). Age-related changes in liver metabolism and antioxidant capacity of laying hens. Poult. Sci..

[4] Wang Y.B., Xu L.P., Sun X.L., Wan X.H., Sun G.R., Jiang R.R., Li W.T., Tian Y.D., Liu X.J., Kang X.T. (2020). Characteristics of the fecal microbiota of high-and low-yield hens and effects of fecal microbiota transplantation on egg production performance. Res. Vet. Sci..

[5] Peebles E., Basenko E., Branton S., Whitmarsh S., Gerard P. (2006). Effects of S6-strain *Mycoplasma gallisepticum* inoculation at 10, 22, or 45 weeks of age on the digestive and reproductive organ characteristics of commercial egg-laying hens. Poult. Sci..

[6] Gu Y.F., Chen Y.P., Jin R., Wang C., Wen C., Zhou Y.M. (2021). A comparison of intestinal integrity, digestive function, and egg quality in laying hens with different ages. Poult. Sci..

[7] Cadenas E., Davies K.J. (2000). Mitochondrial free radical generation, oxidative stress, and aging. Free Radic. Biol. Med..

[8] Estévez M. (2015). Oxidative damage to poultry: From farm to fork. Poult. Sci..

[9] Fellenberg M., Speisky H. (2006). Antioxidants: Their effects on broiler oxidative stress and its meat oxidative stability. World’s Poult. Sci. J..

[10] Sihvo H.-K., Immonen K., Puolanne E. (2014). Myodegeneration with fibrosis and regeneration in the pectoralis major muscle of broilers. Vet. Pathol..

[11] Vercellotti J., St. Angelo A.J., Spanier A.M. (1992). Lipid oxidation in foods: An overview. Lipid Oxidation in Foods.

[12] Gonzalez-Rivas P.A., Chauhan S.S., Ha M., Fegan N., Dunshea F.R., Warner R.D. (2020). Effects of heat stress on animal physiology, metabolism, and meat quality: A review. Meat Sci..

[13] Ismail I., Al-Busadah K., El-Bahr S. (2013). Oxidative stress biomarkers and biochemical profile in broilers chicken fed zinc bacitracin and ascorbic acid under hot climate. Am. J. Biochem. Mol. Biol..

[14] Xing J.Y., Kang L., Hu Y., Xu Q.Y., Zhang N.B., Jiang Y.L. (2009). Effect of dietary betaine supplementation on mRNA expression and promoter CpG methylation of lipoprotein lipase gene in laying hens. J. Poult. Sci..

[15] Shini A., Shini S., Bryden W. (2019). Fatty liver haemorrhagic syndrome occurrence in laying hens: Impact of production system. Avian Pathol..

[16] Ding H.R., Wang J.L., Ren H.Z., Shi X.L. (2018). Lipometabolism and glycometabolism in liver diseases. Biomed. Res. Int..

[17] Kawano Y., Cohen D.E. (2013). Mechanisms of hepatic triglyceride accumulation in non-alcoholic fatty liver disease. J. Gastroenterol..

[18] Freimer D., Yang T.T., Ho T.C., Tymofiyeva O., Leung C. (2022). The gut microbiota, HPA axis, and brain in adolescent-onset depression: Probiotics as a novel treatment. Brain Behav. Immun. Health.

[19] Senchukova M.A. (2023). Microbiota of the gastrointestinal tract: Friend or foe?. World J. Gastroenterol..

[20] Obianwuna U.E., Agbai Kalu N., Wang J., Zhang H.J., Qi G.H., Qiu K., Wu S.G. (2023). Recent trends on mitigative effect of probiotics on oxidative-stress-induced gut dysfunction in broilers under necrotic enteritis challenge: A review. Antioxidants.

[21] Zhang H.Y., Wang Z.Y., Wang G.Q., Song X., Qian Y.Y., Liao Z., Sui L., Ai L.Z., Xia Y.J. (2023). Understanding the connection between gut homeostasis and psychological stress. J. Nutr..

[22] Wu G.P., Li Z.H., Zheng Y., Zhang Y.H., Liu L., Gong D.Q., Geng T.Y. (2022). Supplementing cholamine to diet lowers laying rate by promoting liver fat deposition and altering intestinal microflora in laying hens. Poult. Sci..

[23] Liu M., Kang Z.Y., Cao X.K., Jiao H.C., Wang X.J., Zhao J.P., Lin H. (2024). Prevotella and succinate treatments altered gut microbiota, increased laying performance, and suppressed hepatic lipid accumulation in laying hens. J. Anim. Sci. Biotechnol..

[24] Ejaz A., Wu D.Y., Kwan P., Meydani M. (2009). Curcumin inhibits adipogenesis in 3T3-L1 adipocytes and angiogenesis and obesity in C57/BL mice. J. Nutr..

[25] González-Castejón M., Rodriguez-Casado A. (2011). Dietary phytochemicals and their potential effects on obesity: A review. Pharmacol. Res..

[26] Sakuma S., Sumida M., Endoh Y., Kurita A., Yamaguchi A., Watanabe T., Kohda T., Tsukiyama Y., Fujimoto Y. (2017). Curcumin inhibits adipogenesis induced by benzyl butyl phthalate in 3T3-L1 cells. Toxicol. Appl. Pharmacol..

[27] Manach C., Mazur A., Scalbert A. (2005). Polyphenols and prevention of cardiovascular diseases. Curr. Opin. Lipidol..

[28] Jang H.J., Kim S.S., Oh M.Y., Lee H.H., Han D.J. (2018). Quercetin Enhanced the Function of Mouse Islets. Transplantation.

[29] Yang S., Huo M., Su Z., Wang F., Zhang Y., Zhong C., Shi Y. (2024). The impact of dietary supplementation of Quercetagetin on growth, antioxidant capacity, and gut microbiota of diquat-challenged broilers. Front. Microbiol..

[30] Zheng H.-Q., Tian H.-L., Zhu X.-P., Yin B.-Y., Li Y.-Z. (2024). Effects of quercetagetin on growth performance, diarrhea rate, and immunity of piglets. Feed. Res..

[31] National Research Council (1994). Nutrient Requirements of Poultry.

[32] (2004). Feeding Standard of Chicken.

[33] Zhang T., Li Y.H., Miao M., Jiang B. (2011). Purification and characterisation of a new antioxidant peptide from chickpea (*Cicer arietium* L.) protein hydrolysates. Food Chem..

[34] Klompong V., Benjakul S., Kantachote D., Shahidi F. (2007). Antioxidative activity and functional properties of protein hydrolysate of yellow stripe trevally (*Selaroides leptolepis*) as influenced by the degree of hydrolysis and enzyme type. Food Chem..

[35] Julian R.J. (2005). Production and growth related disorders and other metabolic diseases of poultry—A review. Vet. J..

[36] Jiang J.L., Qi L.N., Dai H.J., Hu C.H., Lv Z.P., Wei Q.W., Shi F.X. (2020). Dietary stevioside supplementation improves laying performance and eggshell quality through increasing estrogen synthesis, calcium level and antioxidant capacity of reproductive organs in aged breeder hens. Anim. Feed. Sci. Technol..

[37] Liang X.X., Fu Y.W., Niu K.M., Zhai Z.Y., Shi H.X., Wang R.X., Yin Y.L. (2023). Dietary *Eucommia ulmoides* leaf extract improves laying performance by altering serum metabolic profiles and gut bacteria in aged laying hens. Anim. Nutr..

[38] Amevor F.K., Cui Z.F., Ning Z.F., Du X.X., Jin N.N., Shu G., Deng X., Zhu Q., Tian Y.F., Li D.Y. (2021). Synergistic effects of quercetin and vitamin E on egg production, egg quality, and immunity in aging breeder hens. Poult. Sci..

[39] Zhao R.Q., Wang Y.J., Zhou Y.C., Ni Y.D., Lu L.Z., Grossmann R., Chen J. (2004). Dietary daidzein influences laying performance of ducks (*Anas platyrhynchos*) and early post-hatch growth of their hatchlings by modulating gene expression. Comp. Biochem. Physiol. Part A Mol. Integr. Physiol..

[40] Yao J., Zhang J., Hou J.F. (2007). Effects of ipriflavone on caged layer bone metabolism in vitro and in vivo. Poult. Sci..

[41] Van Eerden E., Van Den Brand H., Heetkamp M., Decuypere E., Kemp B. (2006). Energy partitioning and thyroid hormone levels during salmonella enteritidis infections in pullets with high or low residual feed intake. Poult. Sci..

[42] Koenig M., Hahn G., Damme K., Schmutz M. (2012). Utilization of laying-type cockerels as “coquelets”: Influence of genotype and diet characteristics on growth performance and carcass composition. Eur. Poult. Sci..

[43] Simitzis P., Spanou D., Glastra N., Goliomytis M. (2018). Impact of dietary quercetin on laying hen performance, egg quality and yolk oxidative stability. Anim. Feed. Sci. Technol..

[44] Reis J.H., Gebert R.R., Barreta M., Boiago M.M., Souza C.F., Baldissera M.D., Santos I.D., Wagner R., Laporta L.V., Stefani L.M. (2019). Addition of grape pomace flour in the diet on laying hens in heat stress: Impacts on health and performance as well as the fatty acid profile and total antioxidant capacity in the egg. J. Therm. Biol..

[45] Shang H.M., Zhou H.Z., Yang J.Y., Li R., Song H., Wu H.X. (2018). In vitro and in vivo antioxidant activities of inulin. PLoS ONE.

[46] Shang H.M., Zhang H.X., Guo Y., Wu H.X., Zhang N.Y. (2020). Effects of inulin supplementation in laying hens diet on the antioxidant capacity of refrigerated stored eggs. Int. J. Biol. Macromol..

[47] Surai P.F., Kochish I.I. (2019). Nutritional modulation of the antioxidant capacities in poultry: The case of selenium. Poult. Sci..

[48] Kishawy A.T., Ibrahim D., Roushdy E.M., Moustafa A., Eldemery F., Hussein E.M., Hassan F.A., Elazab S.T., Elabbasy M.T., Kanwal R. (2023). Impact of resveratrol-loaded liposomal nanocarriers on heat-stressed broiler chickens: Effects on performance, sirtuin expression, oxidative stress regulators, and muscle building factors. Front. Vet. Sci..

[49] He L., He T., Farrar S., Ji L.B., Liu T.Y., Ma X. (2017). Antioxidants maintain cellular redox homeostasis by elimination of reactive oxygen species. Cell Physiol. Biochem..

[50] Hu L.F., Wang Y., Ren R.J., Huo H.R., Sun J.H., Li H.M., Zhu Y.Y., Tan Y.Q. (2016). Anti-oxidative stress actions and regulation mechanisms of Keap1-Nrf2/ARE signal pathway. J. Int. Pharm. Res..

[51] Qi M., Liu K., He J. (2022). Alleviation effect of ginsenoside Rg1 in rats with cholestasis by sirt5 pathway. China Pharm..

[52] Chen X.C., Zhang Y.W., Ma W.F., Wang Z.B. (2020). Effects of *Ligustrum lucidum* on egg production, egg quality, and caecal microbiota of hens during the late laying period. Ital. J. Anim. Sci..

[53] Du J.H., Xu M.Y., Wang Y., Lei Z., Yu Z., Li M.Y. (2022). Evaluation of *Taraxacum mongolicum* flavonoids in diets for *Channa argus* based on growth performance, immune responses, apoptosis and antioxidant defense system under lipopolysaccharide stress. Fish. Shellfish Immunol..

[54] Yang S., Jin X., Xu Y.Q., Shi B.L. (2020). Immunomodulatory effect of flavonoids on broilers and its mechanism. Chin. J. Anim. Nutr..

[55] Lu C.C., Fan G.X., Wang D.Y. (2020). Akebia Saponin D ameliorated kidney injury and exerted anti-inflammatory and anti-apoptotic effects in diabetic nephropathy by activation of NRF2/HO-1 and inhibition of NF-KB pathway. Int. Immunopharmacol..

[56] Gao X.N., Liu P., Wu C., Wang T.C., Liu G.H., Cao H.B., Zhang C.Y., Hu G.L., Guo X.Q. (2019). Effects of fatty liver hemorrhagic syndrome on the AMP-activated protein kinase signaling pathway in laying hens. Poult. Sci..

[57] Trott K., Giannitti F., Rimoldi G., Hill A., Woods L., Barr B., Anderson M., Mete A. (2014). Fatty liver hemorrhagic syndrome in the backyard chicken: A retrospective histopathologic case series. Vet. Pathol..

[58] Hamid H., Zhang J.Y., Li W.X., Liu C., Li M.L., Zhao L.H., Ji C., Ma Q.G. (2019). Interactions between the cecal microbiota and non-alcoholic steatohepatitis using laying hens as the model. Poult. Sci..

[59] Jian H.F., Xu Q.Q., Wang X.M., Liu Y.T., Miao S.S., Li Y., Mou T.M., Dong X.Y., Zou X.T. (2022). Amino acid and fatty acid metabolism disorders trigger oxidative stress and inflammatory response in excessive dietary valine-induced NAFLD of laying hens. Front. Nutr..

[60] Saeed M., Naveed M., Arain M., Arif M., Abd El-Hack M., Alagawany M., Siyal F., Soomro R., Sun C. (2017). Quercetin: Nutritional and beneficial effects in poultry. World’s Poult. Sci. J..

[61] Sohaib M., Butt M.S., Shabbir M.A., Shahid M. (2015). Lipid stability, antioxidant potential and fatty acid composition of broilers breast meat as influenced by quercetin in combination with α-tocopherol enriched diets. Lipids Health Dis..

[62] Guo Y.H., Xu Y.D., Wang D.R., Yang S.H., Song Z.H., Li R., He X. (2024). Dietary silymarin improves performance by altering hepatic lipid metabolism and cecal microbiota function and its metabolites in late laying hens. J. Anim. Sci. Biotechnol..

[63] Fan Y., Pedersen O. (2021). Gut microbiota in human metabolic health and disease. Nat. Rev. Microbiol..

[64] Wastyk H.C., Fragiadakis G.K., Perelman D., Dahan D., Merrill B.D., Yu F.B., Topf M., Gonzalez C.G., Van Treuren W., Han S. (2021). Gut-microbiota-targeted diets modulate human immune status. Cell.

[65] Videnska P., Sedlar K., Lukac M., Faldynova M., Gerzova L., Cejkova D., Sisak F., Rychlik I. (2014). Succession and replacement of bacterial populations in the caecum of egg laying hens over their whole life. PLoS ONE.

[66] Dai D., Qi G.H., Wang J., Zhang H.J., Qiu K., Wu S.G. (2022). Intestinal microbiota of layer hens and its association with egg quality and safety. Poult. Sci..

[67] Panasevich M.R., Meers G.M., Linden M.A., Booth F.W., Perfield J., Fritsche K.L., Wankhade U.D., Chintapalli S.V., Shankar K., Ibdah J. (2018). High-fat, high-fructose, high-cholesterol feeding causes severe NASH and cecal microbiota dysbiosis in juvenile Ossabaw swine. Am. J. Physiol.-Endocrinol. Metab..

[68] Wang Y.M., Chen B., Cao J.M., Huang Y.H., Wang G.X., Peng K., Mo W.Y., Zhao H.X. (2020). Effects of mulberry leaf flavonoids on intestinal mucosal morphology and intestinal flora of *Litopenaeus vannamei*. Chin. J. Anim. Nutr..

[69] Murota K., Nakamura Y., Uehara M. (2018). Flavonoid metabolism: The interaction of metabolites and gut microbiota. Biosci. Biotechnol. Biochem..

